# Supplementary material for: Negative health effects of dental X-rays: A systematic review
Source: PLoS One. 2025 May 29;20(5):e0323808. doi: 10.1371/journal.pone.0323808 (PMC12122044; doi:10.1371/journal.pone.0323808)
Supplement: Attachment — Submitted filename: Response to editor and reviewers.docx [file pone.0323808.s005.docx]

**PONE-D-24-37898 Negative health effects of dental x-rays: a systematic review**

**Respons to Editor and reviewers #1 and #2**

PLOS ONE Journal requirements:

When submitting your revision, we need you to address these additional requirements.

1. Please ensure that your manuscript meets PLOS ONE's style requirements, including

those for file naming. The PLOS ONE style templates can be found at

https://journals.plos.org/plosone/s/file?id=wjVg/PLOSOne_formatting_sample_main_body

.pdf and

https://journals.plos.org/plosone/s/file?id=ba62/PLOSOne_formatting_sample_title_autho

rs_affiliations.pdf

**Reply;** ***We will make sure to follow the journals requirements for PLOS ONE´s style of publication.***

2. We noticed you have some minor occurrence of overlapping text with the following

previous publication(s), which needs to be addressed:

- https://doi.org/10.1080/00016357.2020.180704-

In your revision ensure you cite all your sources (including your own works), and quote or

rephrase any duplicated text outside the methods section. Further consideration is

dependent on these concerns being addressed.

**Reply;** ***Thank you for pointing out the*** ***overlapping of text, this has now been adjusted.***

***The headlines, sublines, tables and figures follow a specific template and is well established for this type of systematic review. Two of the authors are also authors in the previous publication presented and the systematic reviews differ in subject.***

3. Thank you for stating the following financial disclosure: [The study is funded by

Folktandvården/Public Dental Health Care, Stockholms Län AB, Region Stockholm].

At this time, please address the following queries:

a) Please clarify the sourcesa of funding (financial or material support) for your study. List

the grants or organizations that supported your study, including funding received from your

institution.

**Reply;** ***The study is funded by Folktandvården/Public Dental Health Care, Stockholms Län AB, Region Stockholm].***

b) State what role the funders took in the study. If the funders had no role in your study,

please state: “The funders had no role in study design, data collection and analysis,

decision to publish, or preparation of the manuscript.”

**Reply:** ***“The funders had no role in study design, data collection and analysis,***

***decision to publish, or preparation of the manuscript.”***

c) If any authors received a salary from any of your funders, please state which authors and

which funders.

**Reply;** ***Laila Wiklander receives salary from Folktandvården/Public Dental Health Care, Stockholms län AB, Region Stockholm, both as a doctoral student and senior consultant.***

d) If you did not receive any funding for this study, please state: “The authors received no

specific funding for this work.”

**Reply;** ***Folktandvården funded the language revision.***

Please include your amended statements within your cover letter; we will change the online

submission form on your behalf.

**Reply;*The statement has been added to the cover letter.***

4**.** As required by our policy on Data Availability, please ensure your manuscript or

supplementary information includes the following:

A numbered table of all studies identified in the literature search, including those that were

excluded from the analyses.

For every excluded study, the table should list the reason(s) for exclusion.

**Reply; *“S2 Table with 10,949 included records listed with reasons for exclusion” have been added to Supplementary information. The excluded records are listed first -only title and abstract were screened 10,893. 55 included for full text evaluation are at the end of the numbered table (ID nr 10,894-10,949).***

***In table 3 all the excluded records (n=37) are listed under authors, year of publication with reasons for exclusion. Some of the records are letters to editors or a poster or book chapters and cannot be named as studies.***

***In table 4 and 5 the included studies (n=18) are presented for the assessment for risk of bias and divided into either systematic reviews (n=7) or primary studies (n=11) and tools for assessment were ROBIS or ROBINS-E.***

If any of the included studies are unpublished, include a link (URL) to the primary source or

detailed information about how the content can be accessed.

**Reply:** ***There are no unpublished sources included in this review.***

A table of all data extracted from the primary research sources for the systematic review

and/or meta-analysis. The table must include the following information for each study:

Name of data extractors and date of data extraction

Confirmation that the study was eligible to be included in the review.

**Reply*; A table is now added “S3 Table with 11 included primary studies”, names of data extractors and the date for the final compilation. Please see table 6, for the characteristics quality assessment and comments of the include primary studies after the screening by the research group. A short clarifying text has been added to the data extraction section for a date and initials of data extractors. After the updated literature search no studies were added and the table is therefore not revised.***

***A meta-analysis or Synthesis Without Meta-analysis was not possible due to the low quality for this specific review question; see under Section Results and Meta-analysis. Also added a section in the discussion for more clarification.***

All data extracted from each study for the reported systematic review and/or meta-analysis

that would be needed to replicate your analyses.

**Reply;** ***Please see comments above and table 6. No meta-analysis has been presented.***

If data or supporting information were obtained from another source (e.g. correspondence

with the author of the original research article), please provide the source of data and dates

on which the data/information were obtained by your research group.

**Reply:** ***Not applicable, no supporting information has been obtained by correspondence.***

If applicable for your analysis, a table showing the completed risk of bias and

quality/certainty assessments for each study or outcome. Please ensure this is provided

for each domain or parameter assessed. For example, if you used the Cochrane risk-of-

bias tool for randomized trials, provide answers to each of the signalling questions for each

study. If you used GRADE to assess certainty of evidence, provide judgements about each

of the quality of evidence factor. This should be provided for each outcome.

**Reply:** ***Risk of bias assessment is presented in table 4 and 5. GRADE for table 7; A summary of findings for effects exposure in the presented primary studies.***

An explanation of how missing data were handled.

**Reply:** ***We have only included available data and not performed any meta-analysis.***

Reviewers' comments:

Reviewer's Responses to Questions

Comments to the Author

Reviewer #1:

The study is well designed and appropriate consideration has to be given to

not perform meta-analysis given the heterogenity of the included studies. The authors

could have zeroed in on key factors that relate to negative factors as non eof the key words

have emphasized - "adverse effects". Nevertheless, the study is well framed

**Reply;** ***Thank for your comments about keywords; we have revised the keywords and made some changes. Long-Term Adverse Effects have been added.***

Reviewer #2:

1.Keywords: Keywords as per Mesh terms needed. Kindly search keywords on

MeSH on Demand, The MeSH Browser & Direct Browsing of MeSH Hierarchy (Trees). Use

appropriate keywords applying Boolean terms (Please use keywords as per MESH headings

on Medline)

**Reply: *Thank so much for your comments about keywords; we have revised the keywords according to MeSH.***

***The updated Keywords are Radiography, Dental; Pediatric Dentistry; Radiation, Ionizing; Risk Factors; Long Term Adverse Effects; Systematic Review***

2. Incomplete Discussion section & needs to extensively elaborated

***Reply; The discussion section has been revised. The text has been extended with several added paragraphs for greater transparency.***

3. Limitations of this review needs to be mentioned.

**Reply; *Thank you for the comments; This has now been addressed in the discussion section in the elaboration.***

4. Future scope of the review based on the outcomes of total number of studies

synthesized needs to be mentioned

**Reply: *The total number of studies were acceptable but the quality assed to be low. The total number of patients from 4 studies was very low if considered that dental radiographic examinations have been used for over 100 years.***

***In future studies bigger cohorts needs to be included in register-based studies to exclude the risk of recall bias. Confounding factors that can influence the outcome must also be weighed in. Collaborations with other research groups can be a possible way for more statistical power and accurate data and keeping the risk of reporting bias low.***

***The technical advances in health and dental care systems with digitalized records makes it possible to follow children from birth up to 18 years of age and assess the exposures of ionizing radiation. Because of the higher accessibility to radiographic examinations, especially in many developed countries, it is important to include all examinations of ionizing radiation even those with low dose such as intraoral radiographs.***

***Future studies are therefore needed to assess the risk of long-term effects of dental radiography.***

***In the medical health care, there are joined systems for radiographic examinations that can be accessed by private and public clinics and hospitals. It would be great if dentist could have the same possibilities to avoid overexposures especially of children and adolescents that are more sensitive to ionizing radiation.***

Thank you for all your comments and valuable feedback. I truly appreciate it, and I am very grateful for all your insights as they have contributed to a better and clearer description of our work.

Kindest regards

Laila Wiklander
